# Homeopathic drug proving of *Okoubaka aubrevillei*: a randomised placebo-controlled trial

**DOI:** 10.1186/1745-6215-14-96

**Published:** 2013-04-05

**Authors:** Michael Teut, Joern Dahler, Ute Hirschberg, Rainer Luedtke, Henning Albrecht, Claudia M Witt

**Affiliations:** 1 Institute for Social Medicine, Epidemiology and Health Economics, Charité University Medical Center, Luisenstraße 57, 10117, Berlin, Germany; 2 Karl and Veronica Carstens Foundation, Am Deimelsberg 36, 45276, Essen, Germany

**Keywords:** Homeopathic drug proving, Homeopathic pathogenetic trial, *Okoubaka aubrevillei*

## Abstract

**Background:**

Homeopathic drug proving is a basic concept in homeopathy. This study aimed to record symptoms produced by a homeopathic drug compared with placebo.

**Methods:**

This multicentre, randomised, double-blind, placebo-controlled phase 1 trial consisted of a 7-day run-in period, a 5-day intervention period and a 16-day post-intervention observation period. Subjects, investigators and statisticians were blinded for intervention groups and identity of the homeopathic drug. Subjects in the intervention group received *Okoubaka aubrevillei* (potency C12) and subjects in the placebo group received the optically identical sucrose globules. Dosage in both groups was five globules taken five times per day over a maximum period of 5 days. Subjects documented the symptoms they experienced in a semistructured online diary. The primary outcome parameter was the number of characteristic proving symptoms compared with placebo after a period of 3 weeks. Characteristic symptoms were categorised using content analysis. Secondary outcome parameters were the qualitative differences in profiles of characteristic and proving symptoms and the total number of all proving symptoms. The number of symptoms was quantitatively analysed on an intention-to-treat basis using analyses of covariance with the subject’s expectation and baseline values as covariates.

**Results:**

Thirty-one subjects were included (19 Okoubaka and 12 placebo). Data for 29 participants could be analysed. No significant differences in number of characteristic symptoms in both groups were observed between Okoubaka (mean ± standard deviation 5.4 ± 6.0) and placebo (4.9 ± 5.6). The odds ratio for observation of a characteristic symptom was 1.11 (95% confidence interval 0.4 to 3.05, *P* = 0.843). Females and subjects expecting a higher number of symptoms at baseline or feeling more sensitive to homeopathic drugs experienced more characteristic symptoms regardless of allocation. The qualitative analysis showed an inter-coder reliability of 0.69 (95% confidence interval 0.62 to 0.76). The qualitative comparison of symptom profiles was inconclusive.

**Conclusions:**

Combined results of qualitative and quantitative methods did not result in a significant difference of characteristic proving symptoms between *O. aubrevillei* C12 and placebo. The qualitative comparison of the symptom profiles leaves some open questions. The nocebo effect might be a plausible explanation for most of the phenomena observed in this trial.

**Trial registration:**

ClinicalTrials.gov: NCT01061229

## Background

Homeopathic drug proving (HDP) trials, also known as homeopathic pathogenetic trials, are at the foundation of homeopathy and have been conducted for more than 200 years [1-4]. In addition to exposing the toxic effects of the drug and clinical experience, HDPs serve as a key source of information for the homeopathic *Materia Medica*. The purpose of such trials is to test nontoxic levels of a specific substance in healthy volunteers to determine the symptoms this substance stimulates and the types of individuals who may be sensitive to it. The profile of symptoms recorded in an HDP by a group of healthy volunteers serves as a basis for information to find indicators of the drug in sick patients. In HDP, the drug under investigation is administered and the individual response of every volunteer to the application of the substance is described. According to the law of similars, the substance is then used to treat patients with similar symptoms. The clinical experience subsequently shapes the homeopathic drug profile. For 200 years HDPs have been conducted by homeopathic practitioners in the traditional qualitative and explorative HDP design that was described in Hahnemann’s nineteenth-century *Organon der Heilkunst*[5] and other homeopathic classics. In recent years the need to adapt to modern research designs has been realised and an innovative new study methodology for HDP has been developed and tested [6-11].

Within this study we developed a study design methodology and a study protocol that fulfils the criteria of the Good Clinical Practice, Declaration of Helsinki and German drug regulations and also tests its applicability and feasibility in practice [12]. German drug regulation authorities classified this HDP as a phase 1 trial, which can be misleading and has been discussed as controversial [12].

‘Characteristic symptom’ is a very important homeopathic concept to identify the precise homeopathic drug action in homeopathic provings and to find the right homeopathic drug for a patient according to the law of similars (*Organon der Heilkunst* § 153 [5]). Generally, it is considered that a good homeopathic prescription matches the characteristic symptoms of a patient/disease with the characteristic symptoms of a homeopathic drug as derived from a HDP. Homeopaths categorise symptoms as characteristic when they show a high degree of individualising signs. In homeopathy it is believed that the characteristic symptoms in a HDP are specific for the homeopathic action of a drug. Categorising symptoms into characteristic and noncharacteristic is an essential part of homeopathic case-taking and analysis. For a further definition of characteristic symptoms, refer to Table 1. The primary aim of the study was to determine whether the homeopathic drug *Okoubaka aubrevillei* of potency C12 provokes more characteristic homeopathic proving symptoms after 3 weeks compared with a placebo in healthy volunteers.

**Table 1:** Criteria for proving symptoms and characteristic symptoms

**Criteria for proving symptoms**	**Criteria for characteristic symptoms**
	Characteristic symptoms are defined as proving symptoms with a strongly individualistic character:
• New symptoms: the symptom is unfamiliar and has not been observed within the last year or during the run-in period	• Symptoms affecting the whole organism of one or more study participant
• Study participant or study physician classifies the symptom as new or unusual	• Symptoms affecting different organs or organ systems of one or more study participant
• The study physician classifies the symptom as new in his final evaluation	• Symptoms accompanying a variety of other symptoms
• A strong aggravation or modification of present (familiar) symptoms	• Symptoms that occur during the trial that appear strange, peculiar or unique to one or more study participants
• Present familiar symptoms that have disappeared during the proving (cure)	• Familiar symptoms from the past or present that have been cured or strongly alleviated

Secondary aims were to develop and to test a qualitative analysis methodology on which to base a definition for drug-specific (characteristic) symptoms and to compile a profile of characteristic homeopathic proving symptoms of the drug being trialled for therapeutic purposes.

## Methods

### Study design

The HDP trial was conducted as a multicentre, randomised, double-blind, placebo-controlled phase 1 trial. Subjects and investigators were not only blinded to the group allocation process but also to the identity of the drug.

### Subjects

Volunteer medical students or medical doctors were invited to take part in the trial by the investigators via email or telephone.

Subjects were included if they fulfilled the following criteria: medical doctors or medical students, over 18 years of age, not currently being treated for any acute or chronic diseases on the day of inclusion, plus written informed consent.

The following exclusion criteria applied: pregnant women or nursing mothers were excluded, as was anyone who had received homeopathic treatment over the previous 6 weeks, anyone who had participated in another clinical trial during the last 6 months, anyone with a personal or professional dependence on the study physician or sponsor, as well as anyone who had been placed in hospital or another institution by authorities or decree.

### Investigators

The investigators were homeopathic medical doctors with knowledge of HDP and had at least 3 years’ practical experience in homeopathic therapy. All investigators were required to have completed a 2-day certified and standardised investigator training programme.

### Ethics and consent

All subjects provided written informed consent prior to the inclusion. Information about the trial was provided during one-on-one interviews with the help of a written brochure for study subjects. The study was approved by the Berlin Ethics Committee (Landesamt für Gesundheit und Soziales Berlin) on 17 August 2009 (Reference: ZS EK 15, 287/09). The trial was registered under ClinicalTrials.gov: Identifier NCT01061229.

### Procedures

The study consisted of a 7-day run-in period (baseline observation), a 5-day intervention period, followed by a 16-day follow-up observational period.

Each study centre consisted of one investigator who supervised between one and three subjects. After having given informed consent, subjects received an initial physical examination, a full-length homeopathic interview of 60 to 120 minutes’ duration and a short instruction on formal and technical aspects of documentation.

The following items were assessed at baseline: age, sex, education (medical student or doctor), history of former chronic disease, former homeopathic treatment, and former participation in an HDP. Subjects and investigators were also asked to rate their expectations about the subjects’ responsiveness to homeopathic drugs as ordinals with four levels, because the authors presume that this could be a factor related to the outcome.

Questions to subject: (A) ’How would you estimate your sensitivity to homeopathic remedies in general?’ – possible answers: strong reaction / reaction / slight reaction / no reaction; and (B) ’How do you expect to react to the homeopathic drug?’ – answers: a very high number of symptoms / many symptoms / low number of symptoms / no symptoms.

Questions to investigators: (A) ’How would you estimate your subject’s sensitivity to homeopathic remedies in general?’ – response options same as above; and (B) ’What is your expectation about your subject’s reaction to the homeopathic drug?’ – response options same as above.

After starting the trial, subjects were required to document any new or uncommon symptoms in a semistructured trial diary daily (head-to-feet structure to be filled in with free text) accessed through a secure Internet connection. The subjects were supervised by the investigators via telephone in short intervals, as is common in HDP.

### Randomisation and treatment allocation

The random list (block randomisation stratified by centre/investigator) was based on the ‘ranuni’ random number generator of the SAS/STAT® software, SAS Institute Inc., Cary, NC, US. Both the centres and subjects were coded by simple numbers unidentifiable by subjects, investigators and other persons. The randomised list was sent to the Charité central pharmacy, which prepared sealed, sequentially numbered boxes containing the study medication and sent them to the centres. When a subject agreed to participate, the investigator opened the lowest numbered box and gave him/her the study medication. Each centre kept a log file of all randomised subjects.

### Intervention

Subjects in the intervention group were instructed to take five globules of the trial drug (potency C12), five times per day for a maximum of 5 days (5 × 5 × 5). The study medication was obtained from DHU (Karlsruhe, Germany), produced according to the Hahnemannian method [13]. Subjects were asked to stop taking the medication, in agreement with their investigator, if they experienced any of a predefined set of proving symptoms (see Table 1). Placebo consisted of pure sucrose globules (DHU) that were not potentiated nor impregnated with alcohol. The administration scheme in the placebo control group was identical to that of the intervention group.

### Outcome parameters

In our trial we decided to base our hypothesis testing strongly on homeopathic rules and decided to use the peculiar, strange and strongly individual (= characteristic) symptoms as the primary outcome parameter.

The primary outcome parameter was the number of characteristic proving symptoms per subject, derived from the qualitative data analysis of the homeopathic proving drug compared with placebo within 3 weeks after the initial dose of the drug or the placebo is administered. Definitions of characteristic symptoms are given in Table 1.

Secondary outcome parameters were the total number of proving symptoms, irrespective of whether they were characteristic or not, and the number of serious adverse events.

Qualitative differences in the profiles of characteristic proving symptoms from the homeopathic drug and the placebo were compared and the inter-coder reliability of the qualitative evaluation of characteristic proving symptoms was calculated.

### Sample size calculation

In this study, an unpaired *t* test with a two-sided level of 5% has a power of 80% to detect a group difference of 20 ± 4 versus 15 ± 4 (mean ± standard deviation) characteristic proving symptoms. Assuming a dropout rate of 20%, 24 subjects have to be available for analysis. As the sample size calculation was based on an estimation of experts, we decided to include up to 30 subjects.

### Analysis

The qualitative analysis of the textual data of the diaries was carried out by two experienced homeopathic doctors using content analysis [14]. Homeopathic definitions for proving symptoms and characteristic symptoms (Table 1) served as predefined categories for the deductive coding processes. For more details and discussion of the analysis process we refer to the publication of our study protocol [12]. The quantitative analysis was carried out by a statistician.

Our main hypotheses to test were as follows:H0: There is no difference between the number of characteristic symptoms provoked by a homeopathic drug in the potency C12 compared with placebo.; H1: There is a difference between the number of characteristic symptoms provoked by a homeopathic drug in the potency C12 compared with placebo.

The main outcome was analysed on an intention-to-treat basis by univariate analyses of covariance, which includes the group (two levels), subject’s expectations (ordinal with four levels) and the respective baseline value (linear) as covariates. From these models we estimated the baseline-adjusted treatment effect and its 95% confidence interval (CI). The reported *P* value was based on a two-sided *t* test within this model, and *P* <0.05 was considered significant. Missing values were multiply imputed according to Rubin’s suggestions [15]. In detail, 20 multiple copies of the original dataset were generated, replacing missing values by a randomly generated value according to the MCMC algorithm. Each copy was analysed as a complete dataset with the abovementioned analyses of covariance model and the results combined appropriately. Sensitivity analyses included some extensions of the statistical model, in this way modelling the centre as a random factor and adding the centre’s expectation (ordinal with four levels) as an additional fixed factor.

Analyses of the secondary outcome parameters relied on the same statistical models, hereby replacing the baseline value of the main outcome parameters by the baseline value of the parameter under consideration. All analyses were performed using SAS/STAT®.

## Results

**Figure 1:**
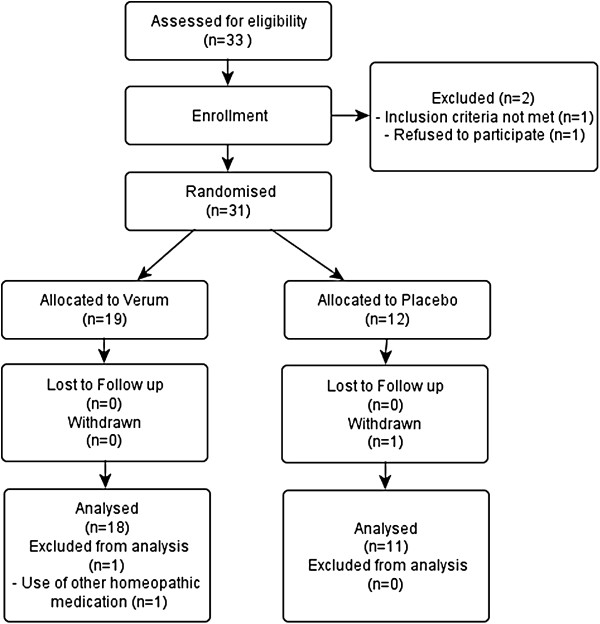
Flowchart of participants.

We included 31 subjects who were supervised by 13 investigators. The flow of participants through the trial is illustrated as a flowchart in Figure 1. The diaries of 29 participants could be analysed and evaluated. One participant withdrew her participation prior to the start of the trial due to acute disease. Another participant had to be excluded as she took additional homeopathic medication during the intervention. There were baseline differences between both groups (Table 2): subjects in the Okoubaka group were on average 7 years younger than those in the placebo group, whereas the placebo group had a higher percentage of medical doctors compared with the Okoubaka group. All subjects in the placebo group estimated that they were sensitive or highly sensitive to homeopathic drugs, whereas this was only the case in 55% of subjects in the Okoubaka group. One-third (36%) of the subjects in the placebo group expected to develop a strong response with many symptoms compared with only 11% in the Okoubaka group.

**Table 2:** Sociodemographic data and characteristics at baseline

	**Placebo (*n*** **= 11)**	**Okoubaka (*n*** **= 18)**
Sociodemographic characteristics		
Age (± standard deviation)	41.1 (± 8.9)	33.9 (± 8.5)
Female (%)	8 (73)	11 (61)
Medical doctors (%)	10 (91)	13 (72)
Medical students (%)	0 (0)	5 (28)
Disease history		
Past chronic disease (%)	6 (54)	9 (50)
Present chronic disease (%)	7 (64)	12 (67)
Experiences		
Experiences in participation in past HDP (%)	2 (18)	3 (16)
Estimation of sensitivity to homeopathic drugs		
Sensitive or highly sensitive		
• Subjects’ estimation (%)	11 (100)	10 (55)
• Investigators’ estimation (%)	10 (91)	15 (73)
Expectation about reaction to homeopathic drug		
Observation of very high numbers / many symptoms		
• Subjects’ estimation (%)	4 (36)	2 (11)
• Investigators’ estimation (%)	9 (82)	10 (56)

HDP, homeopathic drug proving. ^a^One subject was a pharmacist.

No significant differences in the number of characteristic symptoms in both groups were observed. Adjusted means of characteristic symptoms in the Okoubaka group were 5.4 (± 6.0) and in the placebo group 4.9 (± 5.6). The group difference calculated as the odds ratio for observation of a characteristic symptom was 1.11 (95% CI = 0.4 to 3.05), which is not significant (*P* = 0.843, Table 3).

**Table 3:** Outcomes and subgroup analysis

	**Placebo**		***Okoubaka aubrevillei***		
	***n***	**Characteristic symptoms**	***n***	**Characteristic symptoms**	**Significance of group difference, adjusted (*P *value)**
**Outcome**					
Characteristic symptoms	11	4.9 (± 5.6)	18	5.4 (± 6.0)	0.843
Proving symptoms	11	9.6 (± 10.6)	18	8.8 (± 9.6)	0.951
**Subgroup analysis**					
Gender					
Male	3	2.3 (± 3.2)	7	2.3 (± 2.8)	
Female	8	5.9 (± 6.2)	11	7.5 (± 6.7)	
Age					
<35 years	3	4.7 (± 5.6)	13	5.2 (± 5.5)	
≥35 years	7	4.4 (± 6.2)	5	6.2 (± 7.6)	
Sensitivity (subjects’ estimation)^a^					
- Strong reaction / reaction	11	4.9 (± 5.6)	10	5.8 (± 6.7)	
- Slight reaction / no reaction	0	0	7	4.7 (± 5.7)	
Sensitivity (investigators’ estimation)					
- Strong reaction / reaction	10	5.4 (± 5.7)	15	6.3 (± 6.2)	
- Slight reaction / no reaction	1	0	2	1.0 (± 1.4)	
Expected reaction to homeopathic drug (subjects’ estimation)					
- Very high number / many symptoms	4	8.0 (± 7.0)	2	1.5 (± 2.1)	
- Low number / no symptoms	7	3.1 (± 4.2)	15	5.9 (± 6.3)	
Expected reaction to homeopathic drug (investigators’ estimation)					
- *Very high number / many symptoms*	9	6.0 (± 5.7)	10	7.5 (± 6.1)	
- *Low number / no symptoms*	2	0	7	3.0 (± 5.4)	

Characteristic symptoms presented as mean ± standard deviation. ^a^One subject did not rate his expectations.

No significant differences in the number of proving symptoms in both groups were observed. Subjects in the Okoubaka group experienced 8.8 (± 9.6) proving symptoms versus 9.6 (± 10.6) in the control group, the group difference (odds ratio for observation of a proving symptom 1.04, 05% CI = 0.33 to 3.29) was not significant (*P* = 0.951, Table 3).

An analysis of subgroups for sex shows a difference between male and female subjects regardless of group allocation. A mean of 2.3 (placebo: ± 3.2; Okoubaka: ± 2.8) characteristic symptoms were observed in men of both groups, while in women the mean number of characteristic symptoms was much larger with 5.9 (± 6.2) symptoms for placebo and 7.5 (± 6.7) for Okoubaka (Table 3). Generally, subjects expecting a higher number of symptoms at baseline or who felt more sensitive to homeopathic drugs experienced more characteristic symptoms, regardless of group allocation (Table 3).

The two homeopaths who independently coded the text of the diaries for characteristic symptoms reached an inter-coder reliability (Cohen’s kappa) of 0.69 (95% CI = 0.62 to 0.76; before reaching consensus on the symptom categorisation in a final discussion). Table 4 shows the qualitative symptom profiles of Okoubaka and placebo for comparison. In the third row, only symptoms of Okoubaka that were not observed under placebo are listed. These can be compared with a former HDP of *O. aubrevillei* and the clinical homeopathic use in the fourth and fifth rows. The qualitative analysis showed characteristic (highly individual, specific or peculiar) symptoms in both groups. In summary, frequently observed overlapping characteristic symptoms between Okoubaka and placebo were difficulties in concentration, tiredness, headaches, pain in extremities and gastrointestinal disturbances. In the Okoubaka group alone there were general feelings of dryness and burning in multiple parts of the body and feelings of soreness in mucosal membranes and muscles. One subject in the Okoubaka group developed a chronic pharyngitis with vesicular eruptions in the intervention period that lasted for 6 months after the trial. Another subject in the Okoubaka group developed multiple warts. In the placebo group, no symptoms with objective physical and stable pathological changes were observed. Overall there is a high degree of concordance between Okoubaka and placebo and many of these symptoms also show similarities to the symptoms of a former proving and the clinical profile.

**Table 4:** Qualitative symptom profiles of intervention and control groups, former homeopathic drug proving and clinical use

	**Placebo (control)**	***Okoubaka aubrevillei *C12 (intervention)**	**Symptoms only observed in Okoubaka group**	**Profile of *O. aubrevillei *from former homeopathic drug proving **[20]	**Profile of clinical use of *O. aubrevillei ***[19]
Mind	Tranquillity and calmness, feels content although being in trouble, ability to reflect on things from a different perspective.	Concentration deficiency, confusion, disorientation, feeling as if ‘head like in cotton’, ‘like under a bell’, ‘as if drunk’, helplessness, sadness. Oversights, being at loss of words, things fall from the hands, slowing down, fatigue, tiredness, weakness.	Irritability, impatience, helplessness, sadness.	Irritability improves or increases. Discouraged and feeling of being incompetent.	Irritable. Angry. Aggressive and depressive (aggravated before menses), cannot suffer himself, cannot cry. Ambitious, stingy. Concentration weak after influenza/viral infection.
	Senses acute, senses of smell, hearing of voices increased, vision more clear.			Calmness with stressful emotional situation.	
	Tiredness in the day and evening.	Irritability, feeling of mental overload, desire for rest. Impatience.		Fears of aging and poverty.	
				Desire to chew and bite his own inner cheek.	
	Being at loss for words, concentration deficiency, being disorganised, not able to think clearly, feeling of standing beside himself. Mistakes in writing and problems in calculating.	Improved concentration.			
Head	Strong cephalalgias, heading on from the nape outgoing, on both sides, more prominent on the left side, vaguely oppressive, bonnet-like pain from the inside outwards. Aggravated after awakening, the sun, warmth, motoring, beginning of the menses. Ameliorated at 3 p.m., by lying with supported nape, deviance.	Oppressive pains behind eyes and forehead and temples, move to the back of the head. Foggy brained feeling of head. Nausea and loss of appetite. Amelioration by rest, lying, pressure on eyes; aggravation in standing position, walking. Suction-like feeling from the skull cap down to the right side and dizziness.	Foggy brained feeling of head. Headaches and nausea. Suction-like feeling from the skull cap down to the right side.	Congestion in head. Pressure at vertex. Pressure over the left temple as if in a vice.	Awakening at 5 am by cephalalgias. Migraine. Eczema of the scalp. Alopecia.
	Cephalalgias with pain rising up from the nape of neck to the back of the head, and at the same time to the forehead, pulsating and knocking pain in right hemisphere, stitching. Wave-like pulsations, aggravated 12 to 3.30 p.m., in the evening, with beginning of menses, motion; ameliorated by rest, at 4 to 6 p.m. Photosensitivity. Feeling of pressure to eyeballs.				
	Cephalalgias, oppressive, like band feeling around temples, like a nail in the forehead, as if a knife stitches in nasal root and from nasal septum downwards. Feeling of pressure in ears, aggravated in the afternoon, in the evening.				
	Rotary vertigo in the evening, ameliorated while sitting, with sweating and frost feeling in legs.				
Eyes		Dryness, sandy feeling, allergic oculorhinitis.	Dryness, sandy feeling, allergic oculorhinitis.	Swelling beneath the eye. Watery and itching eyes as if allergy season. Sharp pain through the eye. Eye discharge.	Dryness, conjunctivitis. Allergic swelling of eyelids.
Ears		Oozing, itchy exanthema behind the ears.	Oozing, itchy exanthema behind the ears.		Tinnitus, ear pains, deafness.
Nose		Allergic coryza, itching, running or blocked nose, yellow and viscous to liquid mucous, nosebleed.	Allergic coryza, itching, running or blocked nose, yellow and viscous to liquid mucous, nosebleed.	Epistaxis from right nostril.	Chronic sinusitis. Allergic rhinitis.
				Nasal discharge that is clear like with allergy. Sneezing. Aching and burning sinuses.	
Face		Redness and sensations of heat, soreness, dryness, burning.	Sensations of soreness, dryness, burning.	Fever blister on lower lip.	Herpes labialis.
Mouth/Throat	Dryness feeling. Metallic taste. Aphthae.	Feeling of soreness, dryness, redness of the mucosa, chronic pharyngitis with vesicles and wheals (lasting for 6 months), difficulties in swallowing, ameliorated by drinking of hot drinks and eating food, warm foods.	Pharyngitis with vesicles and wheals.	Aphthae on tongue and lower lip that are painful. Boils on the gums. An odour like rotten meat originating from the throat.	Aphthae. Tongue whitish [?], with dental impressions. Suppurations of roots of teeth. Pains swallowing.
	Tongue: burning, soreness, aphthae, vesicles.		Rough scratching, constrictive feeling of throat.		
	Teeth: pulling pains at incisors, pain of molars in the root area, ameliorated with warm foods and drinks.		Changed taste of foods.	Boils in mouth with a rotten meat odour coming from throat	
	Neuralgic pain of upper and lower tooth row on the right side.	Corrosive burning ‘as if burning liquid runs down the pharynx’, rough scratching, constrictive feeling of throat.		Scratching sore throat on swallowing.	
		Lymph nodes at neck swollen. greenish–brown–yellowish sputum. Changed taste of foods. Dry lips, burning sensations. Whitish fur on tongue.			
Stomach	Slack feeling in the stomach, nausea.	Nausea and cephalalgias, loss of appetite, aggravated by thinking of food, ameliorated by eating.	Nausea and cephalalgias, aggravated by thinking of food. Feeling of pressure on stomach.	Nausea after eating or on waking. Sour stomach and burning during the night. Weakness with vomiting.	Feeling as if a stone is lying in the stomach, ameliorated by warm drinks. Feeling of sickness in the morning, with teeth brushing. Vomiting.
		Feeling of pressure on stomach, piercing, hard, cramping sensation. Aggravation in the evening, by belching, eating and drinking. Amelioration by eating.			
Abdomen	Pains in the lower abdomen, spasmodically with flatulence.	Oppressive pains, ‘as if hand around stomach’. Pains in the right epigastrium. Fist-like starting point of pain in the area of the cholecyst, oppressive, pulling sensation of pain. Amelioration by raising, sitting and stretching. Aggravation by stooped seats.	Oppressive pains, ‘as if hand around stomach’. Pains in the right epigastrium. Fist-like starting point of pain in the area of the cholecyst, oppressive, pulling sensation of pain. Amelioration by raising, sitting and stretching. Aggravation by stooped seats.	Pain in general. Cramping pain. Cramping pain better bending over and worse lying or stretching. Intermittent pounding pain on lower left side.	Stomach aches, flatulence, jaundice with cholestase. Pancreas damage by insecticide-loaded food. Diarrhoea after unacceptable food. Thin, bright, sharply smelling stools.
				Offensive flatus in evening. Stabbing pains during bowel movements. Diarrhoea.	
Urinary tract		Burning in the urethra, aggravated by urination.	Burning in the urethra, aggravated by urination.		
Female genitals	Monthly period with increased bleeding.	Monthly period too early or too late. Dysmenorrhoea with spasmodic pains. Stitching and tense pain of the breasts before menses.	Monthly period too early or too late. Dysmenorrhoea with spasmodic pains. Stitching and tense pain of the breasts before menses.	Delayed menses. Short menses.	Irregular bleedings between menses, premenstrual breast pain.
				Increased sexual desire after menses.	
Respiration, chest, heart	Cough, spasmodically with choking. Aggravated in lying. Sharp, strong pain of right rib curves in the axillary line, aggravated in the morning with the awakening, ameliorated by getting up, walking around, external pressure, massage. Inhaling is difficult.	Palpitations. Pulsations. Pressure feeling and narrow feeling on thorax: ‘As if something heavy lies on the chest’. Burning and heat feeling behind sternum. Aggravated by lying on the left side, by drinking green tea, emotional strain.	Palpitations. Pulsations. Pressure feeling and narrow feeling on thorax: ‘As if something heavy lies on the chest’. Burning and heat feeling behind sternum. Aggravated by lying on the left side.	Bronchial itch with cough.	Allergic rhinitis, asthma.
				Sensation as if lungs overexpanded.	
Back	Gluteal pains on the left side, pulling, oppressive, emitting to left femur, aggravated in the mornings, at noon, while sitting, with walking, distension, pressure, driving car.	Myalgias in the shoulder nape area, burning pain, ameliorated by stretching. Pain in the ileosacral area, aggravated by long sitting, standing position, ameliorated by warmth and motion. Sudden clinching back pains in the crossings of TH 12 to L1, mixture of pressure and blockade, ameliorated by motion, bathing feet in the sea, aggravated by sitting and lying.	Burning myalgias, ameliorated by warmth.		
	Pain at left lower abdomen, ameliorated by position change.				
	Pains lumbar vertebra column and ileosacral, on the right side, stitching, oppressive, ameliorated by bending forward.				
Extremities	Knee pain on the left, aggravated by motion, climbing down stairs. Feeling of distortion and pains in wrists and ankle joints, as if swollen, ameliorated by motion.	Pains and cracks in the knee joint. Sensitive soles. Myalgias, cramps. Feeling of heaviness, drawing, soreness in muscles. Tired feelings.	Myalgias, cramps. Feeling of heaviness, soreness in muscles. Tired feelings.	Aching ankle pain or improvement of aching foot arches. Foot pain. Right knee displacement during sleep causing the tendons to draw up the leg. Aching of calf extending from ankle to knee better stretching.	Oedemas of the fingers and ankles. Pain in finger joints. Thumb joint arthrosis.
Sleep	Fatigue with the awakening, night awakening.	Fatigue with the awakening, night awakening.		Sleep disrupted by waking for extended times.	Tiredness, desire to sleep.
				Brooding thoughts that keep her awake.	
Skin	Vesicular eczema of the hands.	Dryness of the skin. Warts, small pinhead-like warts in soles.	Dryness of the skin. Warts, small pinhead-like warts in soles.	Itchy skin.	Eczemas and exanthemas caused by chemical substances and medicines. Acne.
Body temperature / sweating	Flushes of heat and increased sweating.	Coldness, freezing. Need for warmth, feverish sweating.			
Appetite	Appetite decreased, increased.	Desire for warm food.	Desire for warm food.		
Thirst	Decreased thirst feeling.	Dryness of mouth, desire for warm drinks.	Desire for warm drinks.		

Intervention group, *O. aubrevillei*; control group, placebo.

## Discussion

In both groups, characteristic symptoms were identified by the qualitative analyses. Statistically significant group differences in the numbers of characteristic symptoms could not be detected. A subgroup analysis showed that women and individuals expecting to be sensitive to homeopathic drugs experienced more characteristic symptoms, independent of their group allocation. The qualitative analysis of the characteristic symptoms showed a mixed result: there was a wide range of overlapping symptoms for both groups, but also qualitative reporting of symptoms distinguishing Okoubaka from placebo.

In this study we combined high standards of quantitative trial methodology with qualitative methods. We thus combined deductive qualitative analysis methods (coding of characteristic symptoms and proving symptoms) with modern quantitative statistical analyses to investigate differences in a HDP between the homeopathic remedy *O. aubrevillei* C12 and placebo. We used only highly individual (characteristic) symptoms as the primary outcome parameter.

The strength but also a potential limitation of this study is the combination of a qualitative with a quantitative analysis. It discriminates characteristic symptoms from more usual symptoms, allowing a more precise quantitative analysis, but its quality has, on the contrary, a strong impact on the quantitative results. In our trial the results depended on the coding of two homeopaths, both of them with long-term experience in HDPs. The inter-coder reliability showed a Cohen’s kappa of 0.69. The common standard for a good quality of coding is 0.7. This is the first time inter-coder reliability was calculated in an HDP, and resulted in an acceptable level of concordance between the two coding homeopaths.

Another limitation is the unbalanced randomisation allocation, resulting in a significant difference in numbers of included subjects between Okoubaka and control. This imbalance was due to the block-randomisation design stratified by the centre and to some centres only including one or two subjects. This presents a problem for the qualitative analysis, because the range of potential characteristic symptoms in the placebo can be estimated to be smaller in 12 subjects compared with 19 subjects in the Okoubaka group: more individuals could show a wider range of individual symptoms.

The bark of the West African rainforest tree *O. aubrevillei* was introduced into homeopathy in the 1970s by the homeopathic physician Magdalena Kunst and the pharmacist Wilmar Schwabe, who discovered that Okoubaka bark was used by native African healers against food poisoning. *O. aubrevillei* is a registered homeopathic drug in Germany and is mainly used for the treatment of symptoms of gastrointestinal disturbances, food poisoning and food allergies [16-19]. A former HDP of *O. aubrevillei* was conducted by David Riley [20]. The comparison of the symptom profiles of Okoubaka and placebo groups with the results of the former proving and the clinical use remains inconclusive: there is a high overlap of symptoms throughout all groups but there are also symptoms overlapping only between the Okoubaka group and the results of the earlier proving and the clinical profile (compare Table 4).

In our trial there is a notably high number of characteristic symptoms in both groups. Characteristic symptoms are by definition symptoms individualising the complaints or disease; for example, those symptoms that stand out to the subjects both in quality and intensity, and have not been experienced in quality or intensity by subjects before. Most of our subjects observed pathological symptoms that were different from their usual complaints.

Interestingly, female subjects described characteristic symptoms much more frequently than male subjects. Gender differences in HDP have been highlighted previously. In the 1980s, Stübler observed in his scientific HDP that female subjects had more proving symptoms than males [21].

In our opinion, the results must be interpreted with care and we want to discuss hypothetical explanations.

The profiles of symptoms in both groups show a surprisingly high number of characteristic symptoms. One might argue that these cannot be explained by nonspecific effects alone. In our trial, the characteristic symptoms in both groups were compared qualitatively and subsequently compared with the clinical profile of *O. aubrevillei*. A range of similarities was observed. Similarities in symptom profiles between groups proving homeopathic drug and placebo have already been reported by homeopaths in the past. Jeremy Sherr, who actively re-established HDPs in the 1980s and 1990s, writes in his manual *The Dynamics and Methodology of Homeopathic Provings*: ‘… Furthermore it is interesting to note that placebo proving occasionally seem to produce similar symptoms to the proving symptoms, thus casting further doubt on the use of this medium in proving’ [22]. Walach observed in two more recent HDPs (*Atropa belladonna*, and Cantharis) symptoms that were considered highly specific for the homeopathic drug also in the placebo group [8,10,23]. However, these results were in contrast to another homeopathic proving, where a specific effect was observed [9]. It is plausible that the results of this trial may be explained by the nocebo effect. The nocebo effect is by definition the induction of a symptom perceived as negative by sham treatment and/or by the suggestion of negative expectations [24]. A nocebo response is induced by the subject’s own negative expectations and/or negative suggestions from therapists/clinical staff in the absence of any treatment. Nocebo phenomena are generally explained by Pavlovian conditioning and expectations induced by verbal information and suggestions [24]. In our trial this would mean that there were no specific drug effects at all: subjects experienced nonspecific nocebo symptoms regardless of group allocation. The specific, individual and new symptoms in the qualitative analysis might be explained by expectation, suggestion and conditioning of the subjects or just by chance. The observation that subjects with a high expectation of being sensitive to homeopathic drugs experienced more characteristic symptoms regardless of group allocation would fit this hypothesis. Expectation, former experiences and intensified awareness would explain the development of characteristic symptoms in both groups. Nocebo has only recently received wider attention from scientists and clinicians and the available data are sparse compared with research about placebo phenomena [24]. Sex has recently being discussed as a predictor for nocebo response. In a study with healthy subjects measuring symptoms of nausea caused by a spinning chair, gender differences were found: nausea could be conditioned to a significantly higher degree in women, whereas the effects of expectancy were more prominent in men, but to a lesser degree [24,25]. It is also known from nocebo research that patients specify more adverse effects of their medication when checking off a standardised list of symptoms compared with being asked to report spontaneously [26]. Moreover, the way in which trials record adverse events influences the rate of adverse reactions substantially. This might have been the case in our trial, which used semistructured head-to-feet documentation online to be filled with free text symptom descriptions by subjects. The phenomenon of developing homeopathic proving symptoms under placebo has already been described. Bayr stated in 1986 in *The British Homeopathic Journal*: ‘The type of change observed during homeopathic drug trials can also be noted following exhibition of placebo. This makes it more difficult to evaluate the results of homeopathic drug tests’ [27]. Bayr proposed to weight symptoms for the statistical analysis according to their homeopathic symptom characteristics. In our analysis we did not follow Bayr’s advice but we used qualitative methods to eliminate noncharacteristic symptoms for the primary statistical analysis. Therefore our outcome parameter is highly concordant with the homeopathic philosophy. However, we found no statistical group differences.

Another explanation might be that the nocebo response is so prominent that potentially specific symptoms of the drug are difficult to observe and are hidden behind a strong background noise. If this hypothesis was true, the nocebo response as background noise would strongly disturb any HDP and would make it extremely difficult to identify specific symptoms. However, by using a qualitative approach to identify only homeopathic characteristic symptoms, our analysis attempted to filter out such background noise.

Nocebo responses are also frequently observed as adverse events in the placebo arm of randomised controlled drug trials and are known to be a confounding factor in clinical drug trials. In migraine trials, for example, they are observed in 20% of patients after placebo treatment [28]. They also account substantially for adverse events in drug trials for fibromyalgia syndrome and painful diabetic peripheral neuropathy [29]. Surprisingly, the observed adverse events in placebo arms of trials with anti-migraine medication corresponded to those of the anti-migraine medication against which the placebo was compared; the same is the case with nocebo symptoms in antidepressant trials [30]. Nocebo symptoms reflect the typical side effects pattern expected in the drug group, being in accordance with the expectation theory of placebo and nocebo effects [31]. Link and colleagues reported a sham herbal trial where 36 healthy college students were informed they would be given either a herbal supplement or a placebo and were provided with an information sheet with a mock list of possible beneficial (for example, cognitive enhancement) and adverse effects of the supplement, but in fact all participants received placebo [32]. Thirty-two of the 36 participants (88.9%) reported symptoms following ingestion of the pill (50% placebo symptoms, 27.8% placebo and nocebo symptoms, 11.1% only nocebo symptoms). The most frequently observed placebo symptoms were clearer thinking (61.1%) and increased mental alertness (52.8%), and the most frequently observed nocebo symptoms were dry mouth (22.2%), fatigue (16.7%) and headaches (8.3%). In our trial the expectation to observe proving symptoms might have been high, as the subjects were all homeopathic experts and familiar with the concept of HDP.

## Conclusions

Combining qualitative and quantitative methods for the primary outcome did not result in a significant difference of characteristic symptoms between *O. aubrevillei* C12 and placebo. The qualitative comparison of the symptom profiles leaves some open questions. The nocebo effect might be a plausible explanation for most of the phenomena observed in this trial.

## Abbreviations

HDP: Homeopathic drug proving.

## Competing interests

The authors declare that they have no competing interests.

## Authors’ contributions

Study concept and design: MT, JD, UH, CMW. Organisation and data management: UH, MT, CMW. Selection of homeopathic drug: HA. Supervision of investigators: MT, JD, UH. Qualitative analysis: MT, JD, UH. Statistical analysis: RL. Analysis and interpretation of data: MT, JD, UH, CWM, RL. Obtained funding: MT, JD, CMW. Drafting of the manuscript: MT, JD, UH CMW. All authors read, commented on and approved the final manuscript.
